# The effectiveness of diabetes self-management education (DSME) on glycemic control among T2DM patients randomized control trial: systematic review and meta-analysis protocol

**DOI:** 10.1007/s40200-020-00584-3

**Published:** 2020-07-11

**Authors:** Bayu Begashaw Bekele, Samuel Negash, Biruk Bogale, Melkamsew Tesfaye, Dawit Getachew, Fekede Weldekidan, Behailu Balcha

**Affiliations:** 1 Department of Public Health, College of Health Sciences, Mizan Tepi University, Mizan Aman Street, 260, Mizan Aman, Ethiopia; 2 Doctoral School of Health Sciences, University of Debrecen, Debrecen, 4028 Hungary; 3 School of Public Health, College of Medicine and Health Sciences, Woliata Sodo University, Sodo, Ethiopia

**Keywords:** DSME, Glycemic control, Meta-analysis, Randomized control trial, T2DM

## Abstract

**Background:**

Type 2 Diabetes mellitus (T2DM) has been a global pandemic resulting in physical, financial and psychosocial crises. Thus, it is important to investigate pooled effectiveness of Diabetes Self-Management Education (DSME) on glycemic control among T2DM patients.

**Objective:**

The aim of this systematic review and meta-analysis is to investigate the association between DSME or Support (DSME/S) and glycemic control among T2DM patients.

**Methods:**

The systematic review and meta-analysis will include studies conducted throughout the world from 2010 to 2019. T2DM patients and their clinical, anthropometric, biomarkers from baseline to end line will be recorded. We will search all relevant articles from five databases namely; Cochrane Library, BioMed Central, MEDLINE (EBSCOhost), MEDLINE/PubMed and SCOPUS. Key terms will be used for questing relevant articles. Further efforts will be made to check quality of studies base on quality assessment instruments. Finally, the report will be made according Preferred Reporting Items for Systematic Review and Meta-analysis (PRISMA) guidelines. Pooled standard mean difference in HbA1c will be used to calculate the effect size between the variables with random effects analysis assumption. Further subgroup analysis will be carried out for assessing the risk difference among groups.

**Conclusion:**

Based on the existing and eligible researches this systematic review and meta-analysis will bring the best evidence on the effectiveness of DSME/S on glycemic control among the T2DM patients. Moreover, the subgroup analysis will inform the effectiveness heterogeneity based on continent, International Diabetes Federation (IDF) region, intervention period, World bank economic classification and glycemic markers used to follow the patients. The proposed review has been registered in the International PROSPERO website with registration number **CRD42020124236.**

**Electronic supplementary material:**

The online version of this article (10.1007/s40200-020-00584-3) contains supplementary material, which is available to authorized users.

## Introduction

Nowadays diabetes mellitus (DM) affecting nearly half a billion (463 million) people worldwide. If this figure left uncontrolled, it would hit exponentially around 700 million in 2045. Direct and indirect expenses for DM treatment was more than 760 US$ in 2019 [1]. This shows that DM has a tremendous negative impact on physical, psychosocial and financial aspects throughout the world.

Diabetes Self-Management Education/Support (DSME) is an ongoing process of facilitating skill, knowledge and ability for diabetes self-care [2]. It has multifaced importance in improving quality of life, clinical outcomes and behavioral changes among patients [3]. According to the reviews, DSME has positive impact on improving quality of life in all domains, reducing cardiovascular risk reduction, macro and microvascular complications of T2DM among patients [4, 5]. But these studies reported that lack of adequate number of randomized control trial (RCT) studies made the conclusion less inferential. Also, these studies targeted developed regions under their methodology. Similarly, other three meta-analysis concluded that personal care of diabetes has positive impact in improving quality of life of patients. However, these studies were done among Hispanic [6], from 1999 to 2009 [7] and 1980 to 1999 [8]. From these perspectives, the existing reviews are outdated and limited to particular settings in the globe. Among sixteen systematic review and meta-analysis conducted before, none of them included studies from lower and middle income countries (LMICs) specifically Africa [6–21]. After those systematic review and meta-analysis findings, original studies were conducted in Nigeria, Egypt, Ethiopia, and South Africa which met the inclusion criteria [22–24]. Therefore, it is very vital to assess pooled effectiveness of DSME on glycemic control after including the recent studies. This is one of the bold gaps we are trying to fill in this systematic review and meta-analysis (Table 1).

**Table 1:** Summary of existing systematic reviews and meta-analysis on DSME effectiveness and glycaemic control.

S.no	Author	Title	Year of publication	Study setting and time bound	Only Randomized controlled studies included	Number of studies included in meta-analysis	Intervention	Assessment of risk of bias	Finding and conclusion
1.	J. Aquino et al. [10]	Effectiveness of individual strategies for the empowerment of patients with diabetes mellitus: A systematic review with meta-analysis.	2018	Global	Yes	11	Individual empowerment strategies	Yes	Individual strategies for DM empowerment were not effective in reducing HbA1c
2.	S. Mohsen [18]	Health education via mobile text messaging for glycemic control in adults with T2DM: a systematic review and meta-analysis	2014	Global	Yes	10	Text -messaging using a cell phone	No	Mobile short message services improve glycemia among diabetic patients.
3.	S. Norris [8]	Self-management education for adults with type 2 diabetes: a meta-analysis of the effect on glycemic control.	2002	Global studies from 1980 to 1999	Yes	31	Self-management education	No	Self-management education improves HbA1c levels secondary to close follow up
4.	E. Heitkemper [11]	Do health information technology self-management interventions improve glycemic control in medically underserved adults with diabetes? A systematic review and meta-analysis.	2017	Global and no time bound	Yes	13	The effect of health information technology (HIT) diabetes self-management education (DSME)	Yes	HIT supported DSME can potentially improve glycemic level among underserved diabetic adults.
5.	Tiffany L. [12]	Meta-analysis of randomized educational and behavioral interventions in type 2 diabetes	2003	Global and 1966–1999	Yes	18	Educational and behavioral interventions	No	Educational and behavioral interventions (non-drug) in type 2 diabetes have produced modest improvements in glycemic control
6.	SE Ellis [13]	Diabetes patient education: a meta-analysis and meta-regression	2004	Global and from 1990 to 2000	Yes	25	Diabetes education	No	Patient education interventions modestly improve glycemic control in adults with diabetes
7.	AT Schultz [14]	Components of interventions that improve transitions to adult care for adolescents with type 1 diabetes	2016	Global and no time bound	No	4	Transition programs	Yes	Transition interventions may be effective in maintaining glycemic control and reducing diabetic ketoacidosis episodes post transition
8.	Kingshuk Pal [15]	Computer-based interventions to improve self-management in adults with type 2 diabetes: a systematic review and meta-analysis	2014	Global and studies till November 2011	Yes	16	Computer-based diabetes self-management interventions	Yes	Computer-based diabetes self-management interventions has a minimal positive effect on blood glucose control
9.	Pirbaglou, M [16]	Personal Health Coaching as a Type 2 Diabetes Mellitus Self-Management Strategy: A Systematic Review and Meta-Analysis of Randomized Controlled Trials	2018	Global and studies from January 1990 and September 2017	Yes	16	Personal Health Coaching	Yes	Personal health coaching was effective in reducing HbA1c level among T2DM patients.
10.	Pillay J [17]	Behavioral Programs for Type 2 Diabetes Mellitus: A Systematic Review and Network Meta-analysis	2015	Global and studies done from 1993 to 2015	Yes	132 on T2DM and 34 on T1DM	Behavioral programs provision	Yes	Providing behavioral programs have good effect on blood glucose as the time of intervention is long.
11.	M Saffari [18]	Health education via mobile text messaging for glycemic control in adults with type 2 diabetes: a systematic review and meta-analysis.	2014	Global and studies done from January 2003 to November 2013	Yes	10	Mobile text SMS assisted health education	Yes	Mobile text SMS has a significant effect on controlling glycemic level among T2DM patients.
12.	I Ricci-Cabello [19]	Characteristics and effectiveness of diabetes self-management educational programs targeted to racial/ethnic minority groups: a systematic review, meta-analysis and meta-regression	2014	OECD^a^ countries and studies conducted till November 2012	No	20	Diabetes self-management educational	Yes	DSME has a positive outcome in improving knowledge and skill, clinical outcomes among ethnically minority T2DM patients.
13.	Da Tao [20]	Effects of consumer-oriented health information technologies in diabetes management over time: a systematic review and meta-analysis of randomized controlled trials	2017	Global and studies done till 2016	Yes	80	Consumer-oriented health information technologies (CHIT)	Yes	CHIT has good outcome in glycemic control among diabetic patients
14.	Tshiananga, JKT [7]	The effect of nurse-led diabetes self-management education on glycosylated hemoglobin and cardiovascular risk factors: a meta-analysis.	2012	Global 1999 to 2009	Yes	34	Nurse-led diabetes self-management education	Yes	Nurse-led diabetes self-management education was effective in improving glycemic control.
15.	Sherifali D [21]	Evaluating the Effect of a Diabetes Health Coach in Individuals with Type 2 Diabetes	2016	Global and studies conducted till January 2015	Yes	8	Diabetes Health Coach	Yes	Diabetes Health Coach intervention was found to be effective in glycemic control among T2DM patients.
16.	Ferguson, S [6]	Does diabetes self-management education in conjunction with primary care improve glycemic control in Hispanic patients? a systematic review and meta-analysis	2015	Hispanic adults and studies till August 2014	Yes	11	Diabetes Self-Management Education	Yes	DSME was effective in improving glycemic control in Hispanic adults with T2DM

^a^Organisation for Economic Co-operation and Development

On the other hand, we did not find any ongoing systematic review and meta-analysis considered to investigate the effectiveness of DSME/S on glycemic control among T2DM patients. Moreover, International Diabetes Federation (IDF) region, World Bank economic classification, study design, follow up period, and the glycemic markers used to determine T2DM could result in the heterogeneity of the effectiveness. Furthermore, investigating the pool standard mean difference (SMD) in glycated hemoglobin (HbA1c) among intervention and control group will signal early scaling up of the intervention for resource limited settings.

Therefore, we aimed to carry out a subgroup analysis for assessing the pooled effect of DSME/S on glycemic control under consideration of both LMICs and high-income countries. This systematic review and meta-analysis will be important in delivering secondary and tertial prevention for T2DM patients. Also, it will bring a vital information for reducing sufferings and improving quality of life through cost-effective approach in resource constrained settings.

## Methods

The proposed systematic review and meta-analysis has been registered in the International PROSPERO website with registration number CRD42020124236 [25].

### Inclusion and exclusion criteria

#### Type of participants

The criteria for inclusion will be primary studies reported glycemic control based on HbA1c among T2DM patients using Randomized control trial (RCT) design. While studies conducted/reported Gestational or pregnancy and/or Type 1 diabetes mellitus, patients with known history of diabetes, high risk groups like individuals with HIV/AIDS, malignancy, hepatitis, Tuberculosis or any related morbidity will be excluded.

#### Exposure variable

This systematic review and meta-analysis will consider studies those targeted DSME as an intervention for glycemic control.

#### Outcome variable

This systematic review and meta-analysis will focus on studies ascertained blood glucose level. This will be defined glycemic control algorithm that patients with a HbA1C <7% were considered to have good glycemic control. Conversely, those patients HbA1C ≥7% to have poor glycemic control [26]. This cutoff points of HbA1c may vary to some extent from studies to studies. Secondary interest of the study will be the pooled mean differences in the anthropometric and clinical biomarkers.

#### Types of study

This systematic review and meta-analysis will include only RCT in the analysis.

#### Study context and period

This systematic review and meta-analysis will focus on the pertinent previous findings conducted in the seven continents from January 01, 2010 to 31st December 2019.

### Search strategy

Searching for will be carried to find all relevant articles from accessible and the most useful databases both electronically and manually. Electronic databases namely; Cochrane Library, BioMed Central, MEDLINE (EBSCOhost), MEDLINE/PubMed and SCOPUS will be used to search relevant articles. Hand searches will be applied for grey and unpublished researches from Google, Google Scholar, and WHO websites. Key terms will be used to search for articles. These Key terms will be diabetes self-management education, diabetes self-management education and support, DSMES, DSME, diabetes, diabetes mellitus type 2, type 2 diabetes mellitus, type II diabetes mellitus, T2DM, impaired glucose tolerance, NIDDM, Noninsulin Dependent Diabetes Mellitus, outcome, effectiveness, randomized control trial, and RCT.

#### Methodological quality assessment

Four independent reviewers (BBB, SN, Bogale B, Balcha B, DG & MT) will make all effort to check quality of studies based on quality assessment instruments. The Jadad scale (also known as the Oxford quality scoring system) will be used. It is the standard method for evaluating RCTs and consists of three items: randomization, blinding and description of patients’ withdrawals/ dropouts [27]. Any disagreement that arise among the reviewers will be solved thorough discussion or with third reviewer (FW). Authors of the articles will be contacted to find missing full text articles.

### Data extraction

Data will be extracted from included articles in the review using standardized data extraction tool from JBI-MAStARI (see Appendix I). The extracted data will include first author, year, baseline and end line sample size of intervention and control group, baseline and end line intervention and control group mean HbA1c, country, settings, WB economic classification, IDF region, intervention provider and main outcomes those are pertinent to the review question.

### Data synthesis

Initially all quantitative data will be abstracted to Microsoft excel. Then it will be exported into STATA version 14 for pooled in meta-analysis. The heterogeneity following either methodological or clinical among the studies will be assessed using intuitive Index (I^2^). Pooled standard mean HbA1c difference will be used to calculate the effectiveness of DSME on glycemic control among T2DM patients. Metaregression will be made for extraneous factors affecting the association. The publication bias will also be assessed using funnel plot. Both egger and Begg regression tests will be done for the existence of evidence of substantial publication bias for the analysis between DSME and mean glycated hemoglobin (*P* < 0.05 for both tests). Random effect analysis will be considered in the analysis.

## Results

Finally, the report will be made according preferred reporting Items for systematic review and Meta-analysis (PRISMA) [28].

## Discussion

By showing a major paucity of the existing reviews and we started to carry to the current systematic review and meta-analysis. This systematic review and meta-analysis will focus on only RCT studies on the effectiveness of DSME on glycemic control regardless of geographic and economic classification conducted from 2010 to 2019. The final result would help to combat against the complications of diabetes and improving the quality of life particularly LMICs. The main reason and impression are to investigate the DSME approach effectiveness is whether vary by IDF regions, economic levels, follow up periods, education provider.

However, the would be few pitfalls in our way while doing this systematic review and meta-analysis. For instance, inclusion of only English language published results, non-health information technology assisted studies will result in low number of studies. Also excluding studies used FPG as a glycemic marker for glucose control among T2DM patients in some of studies would limit the number of studies included in the analysis.

## Conclusion

The planned systematic review and meta-analysis will investigate the effectiveness of DSME on glycemic control by calculating then pooled mean difference in HbA1c among T2DM patients.

## Electronic supplementary material

ESM 1 (DOCX 198 kb)

## Data Availability

Any data will be available from the first author and corresponding up on request.

## Abbreviations

ADA: American Diabetes Association
DSME: diabetes self-management education
FPG: Fasting plasma glucose
HbA1c: glycated hemoglobin
HIT: Health Information Technology
IDF: International Diabetes Federation
LMICs: Lower- and middle-income countries
NIDDM: Noninsulin Dependent Diabetes mellitus
RCT: randomized control trial
SMD: standard mean difference
T2DM: Type 2 Diabetes mellitus
WHO: World Health Organization
