# Supplementary material for: The effectiveness of diabetes self-management education (DSME) on glycemic control among T2DM patients randomized control trial: systematic review and meta-analysis protocol
Source: J Diabetes Metab Disord. 2020 Jul 11;19(2):1631–7. doi: 10.1007/s40200-020-00584-3 (PMC7843690; doi:10.1007/s40200-020-00584-3)
Supplement: Supplementary file 1 — (DOCX 198 kb) [file 40200_2020_584_MOESM1_ESM.docx]

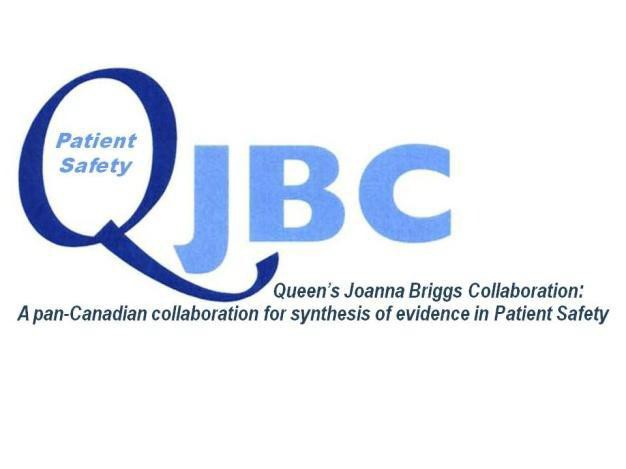


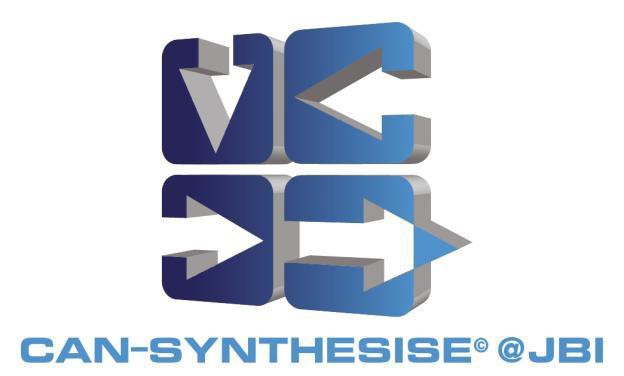


CAN-SYNTHESIZE is a quick reference resource to guide the use of the Joanna Briggs Institute methodology of synthesis*

Queen’s Joanna Briggs Collaboration Version 4.0 January 26, 2015

**for full details please see Joanna Briggs Institute Reviewers’ Manual*


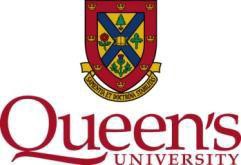

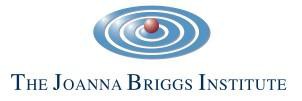


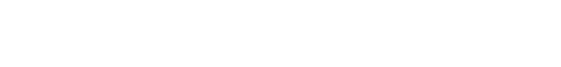


© Christina M. Godfrey and Margaret B. Harrison

© Joanna Briggs Institute


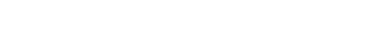


Systematic Review Methodology

**Table of Contents**

Appendix 1: Search strategy decision tree template 2

[Appendix 2: JBI Critical Appraisal for Experimental Studies 3](#_TOC_250006)

[Appendix 3: JBI Critical Appraisal Checklist for Descriptive/ Case Series 4](#_TOC_250005)

[Appendix 4: JBI Critical Appraisal Checklist for Comparable Cohort/Case Control 5](#_TOC_250005)

[Appendix 5: JBI Data Extraction Form for Experimental/Observational Studies 6](#_TOC_250004)

[Appendix 6: JBI Critical Appraisal Checklist for Narrative, Expert Opinion & Text 8](#_TOC_250003)

Appendix 7: JBI QARI Critical Appraisal Checklist for Interpretive & Critical Research …... 9 Appendix 8: JBI Critical Appraisal Checklist for Systematic Reviews 10

[Appendix 9: Data Extraction Template for Qualitative Evidence 11](#_TOC_250002)

[Appendix 10: Extraction of Study Findings Template – for Qualitative Evidence 12](#_TOC_250001)

Appendix 11: JBI Protocol Template 13

Appendix 12: JBI Final Report Template 17

[References 22](#_TOC_250000)

© Christina M. Godfrey and Margaret B. Harrison 1


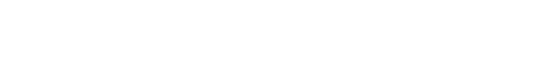

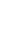


Appendix 1: Search strategy decision tree template^1^

**PRISMA 2009 Flow Diagram**


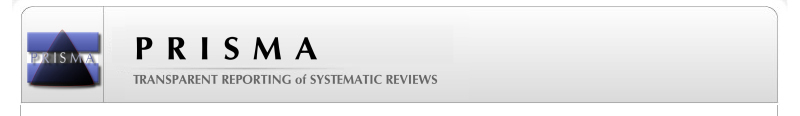


Studies included in quantitative synthesis (meta-analysis)
(n = )

Studies included in qualitative synthesis
(n = )

Full-text articles excluded, with reasons
(n = )

Full-text articles assessed for eligibility
(n = )

Records excluded
(n = )

Records screened
(n = )

Records after duplicates removed
(n = )

Additional records identified through other sources
(n = )

Records identified through database searching
(n = )

## Included

## Identification

## Screening

## Eligibility

## Appendix 2: JBI Critical Appraisal for Experimental Studies2

Reviewer Date

Author Year Record Number

|  | Yes | No | Unclear |
| --- | --- | --- | --- |
| 1. Was the assignment to treatment groups truly random? |  |  |  |
| 2. Were participants blinded to treatment allocation? |  |  |  |
| 3. Was allocation to treatment groups concealed from the allocator? |  |  |  |
| 4. Were the outcomes of people who withdrew described and included in the analysis? |  |  |  |
| 5. Were those assessing the outcomes blind to the treatment allocation? |  |  |  |
| 6. Were control and treatment groups comparable at entry? |  |  |  |
| 7. Were groups treated identically other than for the named interventions? |  |  |  |
| 8. Were outcomes measured in the same way for all groups? |  |  |  |
| 9. Were outcomes measured in a reliable way? |  |  |  |
| 10. Was appropriate statistical analysis used? |  |  |  |

Overall appraisal: Include Exclude Seek further info

Comments (including reasons for exclusion):

## Appendix 3: JBI Critical Appraisal Checklist for Comparable Cohort/ Case Control2

Reviewer Date

Author Year Record Number

|  | Yes | No | Unclear |
| --- | --- | --- | --- |
| 1. Is sample representative of patients in the population as a whole? |  |  |  |
| 2. Are the patients at a similar point in the course of their condition/illness? |  |  |  |
| 3. Has bias been minimized in relation to selection of cases and of controls? |  |  |  |
| 4. Are confounding factors identified and strategies to deal with them stated? |  |  |  |
| 5. Are outcomes assessed using objective criteria? |  |  |  |
| 6. Was follow up carried out over a sufficient time period? |  |  |  |
| 7. Were the outcomes of people who withdrew described and included in the analysis? |  |  |  |
| 8. Were outcomes measured in a reliable way? |  |  |  |
| 9. Was appropriate statistical analysis used? |  |  |  |

Overall appraisal: Include Exclude Seek further info

Comments (including reasons for exclusion):

## Appendix 4: JBI Critical Appraisal Checklist for Descriptive/ Case Series2

Reviewer Date

Author Year Record Number

|  | Yes | No | Unclear |
| --- | --- | --- | --- |
| 1. Was study based on a random or pseudo- random sample? |  |  |  |
| 2. Were the criteria for inclusion in the sample clearly defined? |  |  |  |
| 3. Were confounding factors identified and strategies to deal with them stated? |  |  |  |
| 4. Were outcomes assessed using objective criteria? |  |  |  |
| 5. If comparisons are being made, was there sufficient descriptions of the groups? |  |  |  |
| 6. Was follow up carried out over a sufficient time period? |  |  |  |
| 7. Were the outcomes of people who withdrew described and included in the analysis? |  |  |  |
| 8. Were outcomes measured in a reliable way? |  |  |  |
| 9. Was appropriate statistical analysis used? |  |  |  |

Overall appraisal: Include Exclude Seek further info

Comments (including reasons for exclusion):

## Appendix 5: JBI Data Extraction Form for Experimental/Observational Studies2

Reviewer Author Journal

Date Year Record Number

**Study Method** RCT Quasi-RCT Longitudinal

#### Participants

Retrospective Observational Other

Setting Population Sample size

Intervention 1 Intervention 2 Intervention 3

#### Interventions

**Intervention 1:**

**Intervention 2:**

**Intervention 3:**

**Clinical outcome measures**

| Outcome Description | Scale/measure |
| --- | --- |
|  |  |
|  |  |
|  |  |
|  |  |
|  |  |

**Study results**

Dichotomous data

| Outcome | Intervention ( )  number / total number | Intervention ( )  number / total number |
| --- | --- | --- |
|  |  |  |
|  |  |  |
|  |  |  |
|  |  |  |
|  |  |  |

Continuous data

| Outcome | Intervention ( ) mean & SD (number) | Intervention ( ) mean & SD (number) |
| --- | --- | --- |
|  |  |  |
|  |  |  |
|  |  |  |

#### Authors’ conclusions:

**Comments:**

## Appendix 6: JBI Critical Appraisal Checklist for Narrative, Expert Opinion & Text 2

Reviewer Date

Author Year Record Number

|  | Yes | No | Unclear |
| --- | --- | --- | --- |
| 1. Is the source of the opinion clearly identified? |  |  |  |
| 2. Does the source of the opinion have standing in the field of expertise? |  |  |  |
| 3. Are the interests of patients/clients the central focus of the opinion? |  |  |  |
| 4. Is the opinion's basis in logic/experience clearly argued? |  |  |  |
| 5. Is the argument developed analytical? |  |  |  |
| 6. Is there reference to the extant literature/evidence and any incongruence with it logically defended? |  |  |  |
| 7. Is the opinion supported by peers? |  |  |  |

Overall appraisal: Include Exclude Seek further info

Comments (including reasons for exclusion):

## Appendix 7: JBI QARI Critical Appraisal Checklist for Interpretive

**& Critical Research1**

Reviewer Date

Author Year Record Number

|  | Yes | No | Unclear |
| --- | --- | --- | --- |
| 1. Is there a congruity between the stated philosophical perspective and the research methodology? |  |  |  |
| 2. Is there a congruity between the research methodology and the research question or objectives? |  |  |  |
| 3. Is there a congruity between the research methodology and the methods used to collect the data? |  |  |  |
| 4. Is there a congruity between the research methodology and the representation and analysis of data? |  |  |  |
| 5. Is there a congruity between the research methodology and the interpretation of results? |  |  |  |
| 6. Is there a statement locating the researcher culturally or theoretically? |  |  |  |
| 7. Is the influence of the researcher on the research, and vice versa addressed? |  |  |  |
| 8. Are participants, and their voices, adequately represented? |  |  |  |
| 9. Is the research ethical according to current criteria or, for recent studies, is there evidence of ethical approval by an appropriate body? |  |  |  |
| 10. Do the conclusions drawn in the research report flow from the analysis, or interpretation, of the data? |  |  |  |

Overall appraisal: Include Exclude Seek further info

Comments (including reasons for exclusion):

## Appendix 8: JBI Critical Appraisal Checklist for Systematic Reviews2

Reviewer Date

Author Year Record Number

|  |  | Yes | No | Unclear |
| --- | --- | --- | --- | --- |
| 1. | Is the review question clearly and explicitly stated? |  |  |  |
| 2. | Was the search strategy appropriate? |  |  |  |
| 3. | Were the sources of studies adequate? |  |  |  |
| 4. | Were the inclusion criteria appropriate for the review question? |  |  |  |
| 5. | Were the criteria for appraising studies appropriate? |  |  |  |
| 6. | Was critical appraisal conducted by two or more reviewers independently? |  |  |  |
| 7. | Were there methods used to minimise error in data extraction? |  |  |  |
| 8. | Were the methods used to combine studies appropriate? |  |  |  |
| 9. | Were the recommendations supported by the reported data? |  |  |  |
| 10. | Were the specific directives for new research appropriate? |  |  |  |

Overall appraisal: Include Exclude Seek further info

Comments (Including reasons for exclusion)

## Appendix 9: Data Extraction Template for Qualitative Evidence2

| Method |  |
| --- | --- |
| Methodology |  |
| Interventions |  |
| Setting |  |
| Geographical |  |
| Cultural |  |
| Participants |  |
| Data analysis |  |
| Author’s conclusions |  |
| Reviewer’s conclusions |  |

## Appendix 10: Extraction of Study Findings Template – for Qualitative Evidence2

| Finding |  |
| --- | --- |
| Illustration from publication (including page number) |  |
| Evidence | Unequivocal |
|  | Plausible |
|  | Unsupported |
| Category |  |

**Appendix 11: JBI Protocol Template2**

**Review title**

**Reviewers**

Name 1

Name 2

1

2

# Review question/objective

### Background Inclusion criteria

#### Types of participants

The quantitative component of this review will consider studies that include #describe participants#

The qualitative component of this review will consider studies that include #describe participants#

The textual component of this review will consider publications that include #describe participants#

The economic component of this review will consider studies that include #describe participants#

#### Types of intervention(s)/phenomena of interest

The quantitative component of the review will consider studies that evaluate #insert text# The qualitative component of this review will consider studies that investigate #insert text# The textual component of this review will consider publications that describe #insert text# The economic component of this review will consider studies that evaluate #insert text#

#### Types of outcomes

This review will consider studies that include the following outcome measures: #insert text# The economic component of the review will consider studies that include #insert text#

#### Types of studies

The qualitative component of the review will consider studies that focus on qualitative data including, but not limited to, designs such as phenomenology, grounded theory, ethnography, action research and feminist research.

In the absence of research studies, other text such as opinion papers and reports will be considered. #If you wish to include opinion papers and reports select Textual Evidence and the NOTARI analytical Module and then select to insert set text now#.

The textual component of the review will consider expert opinion, discussion papers, position papers and other text.

The economic component of the review will consider cost effectiveness, cost benefit, cost minimisation, cost utility #modify text as appropriate# studies.

The quantitative component of the review will consider both experimental and epidemiological study designs including randomised controlled trials, non-randomised controlled trials, quasi- experimental, before and after studies, prospective and retrospective cohort studies, case control studies and analytical cross sectional studies #modify text as appropriate# for inclusion.

The quantitative component of the review will also consider descriptive epidemiological study designs including case series, individual case reports and descriptive cross sectional studies

#modify text as appropriate# for inclusion.

# Search strategy

The search strategy aims to find both published and unpublished studies. A three-step search strategy will be utilised in this review. An initial limited search of MEDLINE and CINAHL will be undertaken followed by analysis of the text words contained in the title and abstract, and of the index terms used to describe article. A second search using all identified keywords and index terms will then be undertaken across all included databases. Thirdly, the reference list of all identified reports and articles will be searched for additional studies. Studies published in

#insert language(s)# will be considered for inclusion in this review. Studies published #insert dates# will be considered for inclusion in this review.

The databases to be searched include:

#insert text#

The search for unpublished studies will include:

#insert text#

Initial keywords to be used will be:

#insert text#

# Assessment of methodological quality

Quantitative papers selected for retrieval will be assessed by two independent reviewers for methodological validity prior to inclusion in the review using standardised critical appraisal instruments from the Joanna Briggs Institute Meta Analysis of Statistics Assessment and Review Instrument (JBI-MAStARI). Any disagreements that arise between the reviewers will be resolved through discussion, or with a third reviewer.

Qualitative papers selected for retrieval will be assessed by two independent reviewers for methodological validity prior to inclusion in the review using standardised critical appraisal instruments from the Joanna Briggs Institute Qualitative Assessment and Review Instrument (JBI-QARI). Any disagreements that arise between the reviewers will be resolved through discussion, or with a third reviewer.

Textual papers selected for retrieval will be assessed by two independent reviewers for authenticity prior to inclusion in the review using standardised critical appraisal instruments from the Joanna Briggs Institute Narrative, Opinion and Text Assessment and Review Instrument (JBI-NOTARI). Any disagreements that arise between the reviewers will be resolved through discussion, or with a third reviewer.

Economic papers selected for retrieval will be assessed by two independent reviewers for methodological validity prior to inclusion in the review using standardised critical appraisal instruments from the Joanna Briggs Institute Analysis of Cost, Technology and Utilisation Assessment and Review Instrument (JBI-ACTUARI). Any disagreements that arise between the reviewers will be resolved through discussion, or with a third reviewer.

# Data collection

Quantitative data will be extracted from papers included in the review using the standardised data extraction tool from JBI-MAStARI. The data extracted will include specific details about the interventions, populations, study methods and outcomes of significance to the review question and specific objectives.

Qualitative data will be extracted from papers included in the review using the standardised data extraction tool from JBI-QARI. The data extracted will include specific details about the interventions, populations, study methods and outcomes of significance to the review question and specific objectives.

Textual data will be extracted from papers included in the review using the standardised data extraction tool from JBI-NOTARI. The data extracted will include specific details about the interventions, populations, study methods and outcomes of significance to the review question and specific objectives.

Economic data will be extracted from papers included in the review using the standardised data extraction tool from JBI-ACTUARI. The data extracted will include specific details about the interventions, populations, study methods and outcomes of significance to the review question and specific objectives.

# Data synthesis

Qualitative research findings will, where possible be pooled using JBI-QARI. This will involve the aggregation or synthesis of findings to generate a set of statements that represent that aggregation, through assembling the findings rated according to their quality, and categorising these findings on the basis of similarity in meaning. These categories are then subjected to a meta-synthesis in order to produce a single comprehensive set of synthesised findings that can be used as a basis for evidence-based practice. Where textual pooling is not possible the findings will be presented in narrative form. Textual papers will, where possible be pooled using JBI-NOTARI. This will involve the aggregation or synthesis of conclusions to generate a set of statements that represent that aggregation, through assembling and categorising these conclusions on the basis of similarity in meaning. These categories are then subjected to a

meta-synthesis in order to produce a single comprehensive set of synthesised findings that can be used as a basis for evidence-based practice. Where textual pooling is not possible the conclusions will be presented in narrative form.

Economic findings will, where possible, be pooled using JBI-ACTUARI and presented in a tabular summary. Where this is not possible, findings will be presented in narrative form.

Quantitative papers will, where possible be pooled in statistical meta-analysis using JBI- MAStARI. All results will be subject to double data entry. Effect sizes expressed as odds ratio (for categorical data) and weighted mean differences (for continuous data) and their 95% confidence intervals will be calculated for analysis #modify text as appropriate#. Heterogeneity will be assessed statistically using the standard Chi-square and also explored using subgroup analyses based on the different quantitative study designs included in this review. Where statistical pooling is not possible the findings will be presented in narrative form including tables and figures to aid in data presentation where appropriate.

# Conflicts of interest References

## Appendix 12: JBI Final Report Template2

**JBI – Guidelines for Systematic Review Report Writing**

This document is intended to provide authors with a template with which to write a JBI systematic review report. Each section corresponds to headings in the JBI systematic review and includes a short instruction about the section. In some cases an example is additionally provided. The format of your report should follow this template as closely as possible.

# Author details

#### Instruction:

JBI reviews are required to have a minimum of two authors and they should be listed on the review. Affiliation with JBI should also be included under the contact detail section

# Title of Systematic Review

#### Instruction:

The title of the review should be as descriptive as is reasonable and reflect the systematic review type. If the review is examining clinical effectiveness this should be stated in the title. If specific interventions and/or patient outcomes are examined these should also be included in the title. Where possible the setting and target population should be stated.

#### Example:

“The clinical effectiveness of smoking cessation strategies for adults in acute care mental health facilities” “A comparison of the cost effectiveness of hydrocolloids and calcium alginate dressings in the management of individuals in extended care facilities with stage 3 pressure ulcers”.

# Executive Summary

Should be a summary of the review in 500 words or fewer stating the purpose, basic procedures, main findings and principal conclusions of the study. The executive summary should not contain abbreviations or references.

# Background

#### Instruction:

The background should describe the issue under review including the target population, interventions and outcomes that are documented in the literature. The background should be an overview of the main issues. It should provide sufficient detail to justify the conduct of the review and the choice of the various elements such as the interventions and outcomes. It is often as important to justify why elements are not included. In describing the background literature value statements about effect of interventions should be avoided.

#### Example:

“Use of acupuncture is effective in increasing smoking cessation rates in hospitalised patients”. This is what the review will determine. If this type of statement is made it should be clear that it is not the reviewer’s conclusion but that of a third party, such as “Smith indicates that acupuncture is effective in increasing smoking cessation rates in hospitalised patients”.

# Objectives

#### Instruction:

The review objectives must be stated in full. Conventionally a statement of the overall objective should be made and elements of the review then listed as review questions. This section should be as focused as possible and make explicit what the review intends to find out.

#### Example:

To conduct a systematic review to determine the best available evidence related to the post harvest management of STSG donor sites. The specific review questions to be addressed are:

What interventions/dressings used in the management of the STSG donor site are most effective;

in reducing time to healing, in reducing rates of infection,

and in reducing pain levels and promoting comfort?

What interventions/dressings are most effective in managing delayed healing/infection in the split skin graft donor site? What interventions are most effective in managing the healed split skin donor site?

# Criteria for Considering Studies for this Review

### Types of studies

#### Instruction:

This section should flow from the background. Many JBI reviews will have a hierarchy of studies that have been considered. There should be a statement about the target study type and the range of studies that were used if the primary study type was not found.

### Types of participants

#### Instruction:

There should be details about the type of individuals targeted including characteristics (e.g. age range), condition/diagnosis or health care issue (e.g. administration of medication in rural areas and the setting(s) in which these individuals are managed. Again the decisions about the types of participants should have been justified in the background.

### Types of interventions

#### Instruction:

There should be a list of all the interventions examined. In some cases it may be appropriate to list categories of interventions. For example “pharmaceutical and non-pharmaceutical interventions for smoking cessation”. This section should be concise as the background section provides an opportunity to describe the main aspects of the interventions.

### Types of outcome measures

#### Instruction:

There should be a list of the outcome measures considered. The clinical relevance of the outcomes must be considered. The background should provide enough information to justify the outcomes included.

# Search Strategy

#### Instruction:

An overview of the search strategy should be provided. It is usual to undertake a staged approach including initial search, full search and search of reference lists and hand searching. The databases searched should be listed with the time frames included. The initial search terms should be appropriate for the review objectives. If reference lists, grey literature and selective hand searching are used this should be stated. A statement about assessment should be included.

#### Example:

“All studies identified during the database search were assessed for relevance to the review based on the information provided in the title, abstract, and descriptor/MeSH terms. A full report was retrieved for all studies that met the inclusion criteria (see appendix I). Studies identified from reference list searches were assessed for relevance based on the study title.” Reference should be made to the inclusion criteria that should be within a checklist in the appendices.

# Methods of the Review

### Assessment of methodological quality

#### Instruction:

A description of how methodological assessment was managed should include reference to the checklist used by the review team that is included in the appendices.

### Data extraction

#### Instruction:

A description of how data extraction was managed should include reference to the data extraction tool used by the review team that is included in the appendices. In some cases revision of the data extraction tool will occur after the full search has been conducted. A statement should be made to that effect.

### Data synthesis

A description of how data synthesis was managed should be included. Where meta-analysis was been used, the statistical methods and the software used (RevMan or SUMARI) should be described and if appropriate justified. For instance if standard mean differences were used instead of weighted means.

# Review Results

### Description of studies

The type and number of papers identified and the number of papers that were included and excluded should be stated.

### Methodological quality

This should be a summary of the overall quality of the literature identified.

### Results

This section must be organised in a meaningful way based on the objectives of the review and the criteria for considering studies, particularly considering types of interventions and outcomes. The reviewer should comment on the appropriateness of meta-view graphs.

# Discussion

The discussion should include an overview of the results. It should address issues arising from the conduct of the review including limitations and issues arising from the results of the review.

# Conclusions

### Implications for practice

Where possible implications for practice should be detailed, but must be based on the documented results, not author opinion. Where evidence is of a sufficient level, appropriate recommendations should be made. Recommendations must be clear, concise and unambiguous.

### Implications for research

All implications for research must be derived from the results of the review.

# References

#### Instruction:

The references should be appropriate in content and volume and include background references and studies from the initial search. The format must be Vancouver.

# Appendices

#### Instruction:

The appendices should include:

- Critical appraisal form(s)
- Data extraction form(s)
- Table of included studies
- Table of excluded studies with justification for exclusion

These checklists should reflect the types of studies, settings, participants, interventions, and outcomes for the review question posed.

**References**

1. Moher D, Liberati A, Tetzlaff J, Altman DG, The PRISMA Group (2009). *P*referred *R*eporting *I*tems for *S*ystematic Reviews and *M*eta-*A*nalyses: The PRISMA Statement. PLoS Med 6(6): e1000097. doi:10.1371/journal.pmed1000097. For more information, visit [www.prisma-statement.org](http://www.consort-statement.org/).
2. The Joanna Briggs Institute. Joanna Briggs Institute Reviewers’ Manual: 2014 edition. The Joanna Briggs Institute: Adelaide.
